# Reference gene selection for head and neck squamous cell carcinoma gene expression studies

**DOI:** 10.1186/1471-2199-10-78

**Published:** 2009-08-03

**Authors:** Benjamin Lallemant, Alexandre Evrard, Christophe Combescure, Heliette Chapuis, Guillaume Chambon, Caroline Raynal, Christophe Reynaud, Omar Sabra, Dominique Joubert, Frédéric Hollande, Jean-Gabriel Lallemant, Serge Lumbroso, Jean-Paul Brouillet

**Affiliations:** 1 Service d'ORL et Chirurgie Maxillo-faciale, Centre Hospitalier Universitaire de Nîmes, Place du Pr. Robert Debré, 30029 Nîmes Cedex 9, France; 2 Centre National de la Recherche Scientifique, Unité Mixte de Recherche 5203, Institut de Génomique Fonctionnelle, Montpellier F-34094, France; 3 Institut National de la Santé et de la Recherche Médicale, U661 Montpellier F-34094, France; 4 Université Montpellier 1, Montpellier F-34094, France; 5 Laboratoire de Biochimie, Centre Hospitalier Universitaire de Nîmes, Place du Pr. Robert Debré, 30029 Nîmes Cedex 9, France; 6 Service d'Information Médicale et de Biostatistique, Centre Hospitalier Universitaire de Nîmes, Place du Pr. Robert Debré, 30029 Nîmes Cedex 9, France; 7 CRC-Service d'Epidémiologie Clinique, Hôpitaux Universitaires de Genève, Rue Micheli-du-Crest 24, CH-1211 Genève 14, Suisse; 8 Service d'Anatomie et Cyto-pathologie, Centre Hospitalier Universitaire de Nîmes, Place du Pr. Robert Debré, 30029 Nîmes Cedex 9, France

## Abstract

**Background:**

It is no longer adequate to choose reference genes blindly. We present the first study that defines the suitability of 12 reference genes commonly used in cancer studies (*ACT, ALAS, B2M, GAPDH, HMBS, HPRT, KALPHA, RPS18, RPL27, RPS29, SHAD *and *TBP*) for the normalization of quantitative expression data in the field of head and neck squamous cell carcinoma (HNSCC).

**Results:**

Raw expression levels were measured by RT-qPCR in HNSCC and normal matched mucosa of 46 patients. We analyzed the expression stability using geNorm and NormFinder and compared the expression levels between subgroups. In HNSCC and/or normal mucosa, the four best normalization genes were *ALAS, GAPDH, RPS18 *and *SHAD *and the most stable combination of two genes was *GAPDH-SHAD*. We recommend using *KALPHA-TBP *for the study of T1-T2 tumors, *RPL27-SHAD *for T3-T4 tumors, *KALPHA-SHAD *for N0 tumors, and *ALAS-TBP *for N+ tumors. *ACT, B2M, GAPDH, HMBS, HPRT, KALPHA, RPS18, RPS29, SHAD *and *TBP *were slightly misregulated (<1.7-fold) between tumor and normal mucosa but can be used for normalization, depending on the resolution required for the assay.

**Conclusion:**

In the field of HNSCC, this study will guide researchers in selecting the most appropriate reference genes from among 12 potentially suitable reference genes, depending on the specific setting of their experiments.

## Background

RT-qPCR is a simple, fast, cost-effective and sensitive technique that has been extensively used in cancer research. In the field of head and neck squamous cell carcinoma (HNSCC), RT-qPCR has mainly been used to identify gene regulation in tissue from the upper aerodigestive tract induced by conditions such as cancer or drug, alcohol and tobacco use. From a clinical point of view, this approach aims to discover transcriptional alterations that can be used for diagnosis, classification and/or prognosis [1]. Among the pitfalls of this measuring tool, the normalization step is certainly one of the most debated [2]. RT-qPCR normalization procedures have been developed in order to minimize inter-sample variability due to technical artifacts such as flaws in RNA concentration assessment or the handling process, as well as variable retro-transcription efficiency [3,4]. The vast majority of RT-qPCR studies rely on the measurement of internal control genes, called housekeeping genes or reference genes, simultaneously with the genes of interest. Since the reference genes are exposed to the same preparation steps as the genes of interest, this normalization adjusts for differences in amount and quality of starting material [5]. A perfect reference gene should have a steady expression in different tested tissues and should not be regulated by physiological or pathological mechanisms or by external causes. Unfortunately, it has been clearly demonstrated that a universal reference gene does not exist and that even housekeeping gene expression can be influenced by cellular processes like differentiation, cell cycle, and cancer progression, or modulated by external factors such as drugs, radiotherapy and hormonal changes [6-9]. Despite this evidence, which highlights the importance of validating a potential reference gene for each specific experimental condition, most RT-qPCR studies employ arbitrarily selected endogenous genes without proper validation of their presumed stability of expression. This negligence could lead to systematic false measurements and, consequently, to erroneous conclusions [3,10].

The systematic study of the suitability of reference genes for RT-qPCR normalization in the field of HNSCC has thus far been lacking. We thus aimed to test the appropriateness of 12 commonly used reference genes (*ACT, ALAS, B2M, GAPDH, HMBS, HPRT, KALPHA, RPS18, RPL27, RPS29, SHAD *and *TBP*) for RT-qPCR normalization. We evaluated their expression stability in HNSCC and matched normal mucosa and we looked at potential differential regulation between clinically relevant subgroups (tumor versus normal mucosa, T1-T2 versus T2-T3 stages, N0 versus N+ stage). Because the use of at least two reference genes is recommended, we indicate for each tissue subgroup the best combination of two genes that should be privileged [11].

## Results

### Raw Cp values of reference genes

**Figure 1:**
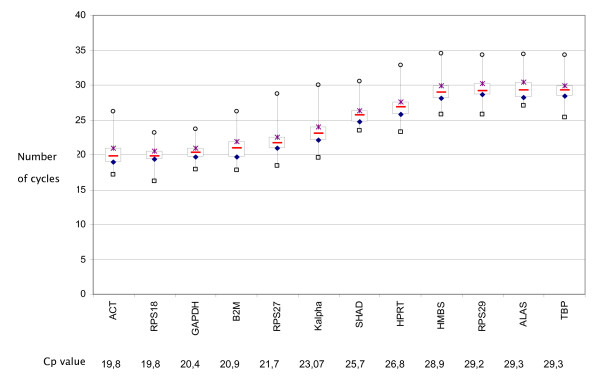
**mRNA expression of 12 reference genes in HNSCC tissue and matched normal mucosa**. Raw Cp values are represented for each gene by a box-plot. The central box represents the interquartile interval, the red line inside the box is the median value, and the extreme values represent the minimum and maximum values. Cp (Crossing point).

The median expression range of the 12 tested genes was calculated from raw Cp values and spanned 19.8 cycles for *ACT *to 29.2 cycles for TBP. As presented in Figure 1, expression levels of *ALAS, HMBS, RPS29 *and *TBP *were low, with median Cp values between 28 and 30 cycles. *HPRT, KALPHA *and *SHAD *displayed intermediate expression levels with median Cp values between 23 and 26 cycles. In contrast, high expression of *ACT, B2M, GAPDH, RPS18 *and *RPL27 *was detected, with Cp values between 19 and 22 cycles. Among the 12 genes, the maximum and minimum expression range was 10.4 cycles for *KALPHA *and 5.8 for *GAPDH*, respectively.

### Reference gene expression stability in the pool of HNSCC plus normal mucosa samples

**Figure 2:**
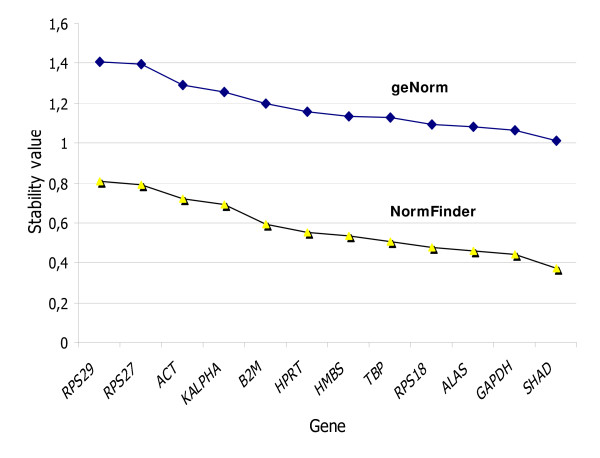
**geNorm and NormFinder stability values of 12 reference genes in 46 HNSCC and normal matched mucosa**. Upper line with rhombus = stability values from geNorm; Lower line with triangles = stability values from NormFinder.

We first studied the inter-sample stability of reference gene expression in the pool of HNSCC plus normal mucosa samples. Using geNorm software, we found that M values for all 12 studied genes were falling below the 1.5 threshold, under which a gene is considered suitable for normalization by this program. The best combination of two genes for normalization was *GAPDH *with *SHAD*, an association that reached a 0.722 M value. As presented in Figure 2, when samples were considered independently using the NormFinder software, we found exactly the same ranking as with geNorm. When tumor and normal tissues were taken into consideration using the NormFinder pairing option, we observed slight modifications in gene stability ranking, but *GAPDH *and *SHAD *remained the two best single normalization genes and their association was still the best combination of two genes, with a 0.066 stability value.

### Reference gene expression stability in HNSCC and normal mucosa separately

We then applied stability tests to HNSCC and normal samples separately in order to identify differences with the whole sample group. In both types of tissue, M values provided by geNorm were still under the 1.5 cut-off for the 12 genes. Interestingly, the four best normalization genes in both groups given by geNorm as well as NormFinder were *ALAS, GAPDH, RPS18 *and *SHAD*. Similarly, the four worst genes were *ACT, KALPHA, RPL27 *and *RPS29*. In the HNSCC group, the best combination of two genes was *GAPDH *and *SHAD *with an M value of 0.732. In the normal mucosa group, the best combination of two genes was *GAPDH *and *RPS18 *with an M value of 0.654, followed by *GAPDH *and *SHAD *with an M value of 0.709.

### Reference gene expression stability in HNSCC subgroups: T and N Stage

More variable results were observed when stability tests were applied to HNSCC subgroups. In the T stage subgroup, both geNorm and NormFinder found *KALPHA *and *TBP *to be the two most stable genes for normalization of T1-T2 tumors, while *RPL27 *and *SHAD *were the most stable for T3-T4 tumors. In the N stage subgroup, both geNorm and NormFinder found *KALPHA *and *SHAD *to be the two most stable genes for N0 tumors and *ALAS *and *TBP *for N+ tumors. It is worth noting that *RPL27 *was considered by geNorm as unsuitable for normalization in T1-T2 and N0 HNSCC subgroups, with an M value >1.5.

### Reference gene expression compared in HNSCC and normal matched mucosa

**Figure 3:**
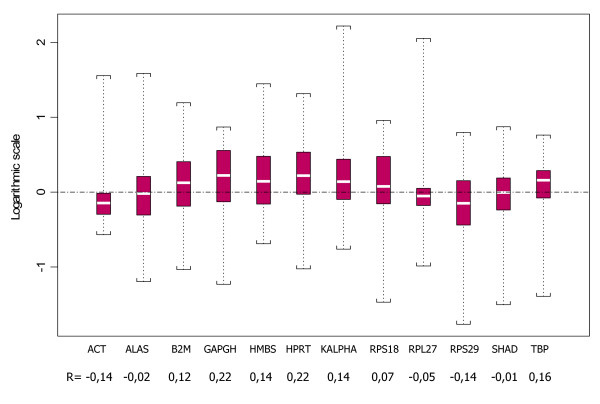
**Expression ratio between HNSCC tissue and normal mucosa in the 12 reference genes**. For estimation of the individual expression of each gene, the expression ratios of paired tissue specimens were calculated (R = HNSCC/normal). The log distribution of these ratios is represented for each gene by a box-plot. The central box represents the interquartile interval, the white line inside the box is the median value, and the extreme values represent the minimum and the maximum.

As geNorm and NormFinder are not able to address the specific issue of inter-group comparison, we statistically evaluated the relative expression levels in HNSCC and normal matched tissue for the 46 patients. As presented in Table 1, relative mRNA expression levels were significantly higher in tumors than in normal samples for *B2M, GAPDH, HMBS, HPRT, KALPHA, RPS18 *and *TBP *(Wilcoxon test for paired data, p < 0.001). These expression levels were lower in tumor than in normal samples for *ACT *and *RPS29 *(Wilcoxon test for paired data, p < 0.05). As presented in Figure 3, it should be noted that the median expression ratio between tumor tissue and matched normal mucosa was very low with a maximum over-expression of 1.4-fold for *ACT *and *RPS29 *and a maximum expression decrease of 1.7-fold for *GAPDH *and *HPRT*.

**Table 1:** Differential expression of candidate reference genes in HNSCC tissue and matched normal mucosa (46 patients)

	**Normal tissue**			***HNSCC tissue***			
**Gene**	**Median**	**Min**	**Max**	***Median***	***Min***	***Max***	P value
**ACT**	**2.302**	0.029	8.642	***1.466***	*0.151*	*27.537*	<0.001
**ALAS**	**2.407**	0.065	8.106	***2.426***	*0.083*	*14.561*	**0.623**
**B2M**	**1.384**	0.062	7.521	***2.148***	*0.112*	*11.168*	0.038
**GADPH**	**2.087**	0.368	5.070	***2.948***	*0.145*	*17.192*	<0.001
**HMBS**	**1.944**	0.045	5.278	***1.989***	*0.238*	*10.071*	0.043
**HPRT**	**1.365**	0.037	3.708	***2.316***	*0.102*	*18.128*	<0.001
**KALPHA**	**1.590**	0.020	5.908	***3.174***	*0.095*	*17.656*	<0.001
**RPS18**	**2.207**	0.258	12.132	***2.690***	*0.093*	*21.544*	0.039
**RPL27**	**2.112**	0.022	8.797	***2.027***	*0.228*	*23.993*	**0.140**
**RPS29**	**2.875**	0.090	22.910	***2.362***	*0.020*	*18.233*	0.015
**SHAD**	**1.612**	0.064	4.218	***1.723***	*0.074*	*13.097*	**0.939**
**TBP**	**1.466**	0.151	27.537	***1.645***	*0.224*	*7.052*	0.010

Median, minimum and maximum relative gene expression ratios of 12 reference genes in HNSCC tissue versus normal tissue are presented with corresponding p-values (Wilcoxon test for paired data). Rescaled values provided by qBase software are presented.

### Comparison of reference gene expression in T1-T2 and T3-T4, and N0 and N+ HNSCC

The relative expression levels of the 12 genes were compared between T1-T2 and T3-T4 and between N0 and N+ HNSCC. No significant difference was shown between these tumor subgroups (Wilcoxon test, p > 0.05).

### Evaluation of the robustness of geNorm results by bootstrap technique

We bootstrapped the geNorm results to assess their robustness (M value, ranking and 1.5 suitability threshold).

For the ranking, the bootstrap results were consistent with those of the original data set. As presented in Table 2, *SHAD *was ranked the first or second most stable gene in 83% of the 10,000 generated bootstrap samples. *GAPDH *remained the second most stable gene with a ranking of second or third in 56% of the cases. It should be noted that the ranking of some genes was highly affected by the bootstrap procedure. For instance, *RPL27 *was ranked the second most stable gene in 15% of the cases, but it was also ranked twelfth and the least stable gene in 38% of the cases. These results underscore the fact that gene ranking is not an absolute means to select the most stable gene, particularly when stability results are not very different, which was the case for our set of 12 genes.

**Table 2:** Ranking of the 12 candidate genes after geNorm bootstrap.

**Rank**	**SHAD**	**GAPDH**	**ALAS**	**RPS18**	**TBP**	**HMBS**	**HPRT**	**B2M**	**Kalpha**	**ACT**	**RPL27**	**RPS29**
**1**	59	2	>1	>1	16	11	0	0	>1	>1	8	0
**2**	24	22	13	4	8	7	>1	0	4	>1	14	0
**3**	8	33	16	14	6	6	>1	>1	4	1	8	0
**4**	5	18	20	31	7	6	1	>1	3	1	3	0
**5**	2	11	18	33	8	10	8	1	4	2	>1	0
**6**	1	7	13	13	9	15	24	6	6	4	>1	0
**7**	>1	4	8	2	8	16	27	19	7	6	0	>1
**8**	>1	1	5	>1	10	17	19	28	8	8	0	>1
**9**	>1	>1	2	>1	7	7	13	20	24	14	2	7
**10**	0	0	>1	0	9	2	5	18	21	27	4	11
**11**	0	0	>1	0	5	>1	>1	7	12	18	18	38
**12**	0	0	0	0	1	0	0	0	2	14	38	43
***Total***	*100%*	*100%*	*100%*	*100%*	*100%*	*100%*	*100%*	*100%*	*100%*	*100%*	*100%*	*100%*

This table presents for each gene the frequencies (in percentage) of ranking between the first and the twelfth position for the 10,000 iterations of geNorm bootstrap.

We observed that median M values (based on the 10,000 iterations) were falling under the 1.5 threshold for all 12 genes. Surprisingly, *RPL27 *(one of the least stable genes) had the best median M value (M = 0.994), whereas *SHAD *(one of the most stable genes) had one of the worst median M value (M = 1.257). We thus decided to analyze whether some genes presented M values above the 1.5 cut-off value in some of the 10,000 bootstrap geNorm results. *RPS 29, TBP, HPRT, SHAD, ACT *and *KALPHA *exhibited M values above this stability threshold in respectively 1%, 5%, 10%, 13%, 15% and 30% of the cases.

## Discussion

The measurement by RT-qPCR of quantitative transcriptional differences between several physiological or pathological conditions is of great interest for clinical applications. The major challenge of this mRNA quantization is the variability introduced at each step of the procedure by experimental errors or approximation and by variable enzymatic activity. To date, the most commonly used strategy to deal with this problem has been the normalization of raw data by at least one internal reference gene, so-called reference gene [3,7,8]. The pitfall of this approach is that no universal reference gene exists since many of them are regulated in several conditions, which may lead to altered findings and wrong experimental conclusions. It has been emphasized that researchers need to choose the most appropriate reference gene for a given tissue or disease, and should therefore prove the suitability of these genes in each specific experimental situation [12,13].

In the field of HNSCC, this study is the first one that aimed to select suitable reference genes for the normalization of RT-qPCR studies based on the analysis of human tissue. A similar approach was employed in other types of cancer (breast, colon, bladder, kidney, prostate), but the present work is particularly notable for the number of selected genes, number of paired samples, rigorous quality control and biostatistical analyses [9,14-19].

Two parameters are key to evaluating the expression stability of reference genes: inter-sample stability and inter-group stability [15,20,21].

First, we assessed the theoretical inter-sample expression stability of the 12 studied genes using two popular, dedicated software programs: geNorm and NormFinder. We used this approach for the sample group treated as a whole, as well as for the clinically relevant subgroups (tumor tissue, normal mucosa, T1-T2 stage, T3-T4 stage, N0 stage, N+ stage), since quantitative expression studies frequently include one of these subgroups to address specific clinical issues. We found that the 12 candidate genes could be considered as stable enough for normalization purposes, except for *RPL27 *in T1-T2 or N0 stage tumors. Moreover, the results given by both software programs were highly comparable. Although the stability values were very close, some of the 12 genes appeared to be more stable than others and should probably be used as a priority (*ALAS, GAPDH, RPS18 *and *SHAD*). Previous data suggested that at least two reference genes should be associated for accurate normalization [11], and our results clearly showed that indeed, the association of two genes dramatically improved the stability values, and that *GAPDH-SHAD *appeared to be the best association for HNSCC studies.

In the literature, more or less sophisticated strategies have been proposed to assess the inter-sample expression stability of reference genes [11,20,22]. Since no universal reference point is available, these methods are all based on averaged cross-comparisons of the expression level of all genes in all measured samples. This principle is certainly relevant but has its own limitations. Indeed, the results greatly depend on the choice of sample and on the genes introduced into the analyses. Moreover, the reliability of results provided by geNorm and NormFinder is questionable, notably because these programs do not include a statistical evaluation of these results. We thus decided to bootstrap the results of geNorm, in order to test their robustness [23,24]. Although the bootstrap results were globally consistent with the original data set, this statistical procedure confirmed that stability values and gene ranking are highly altered by some changes in the original dataset. Moreover, Lyng et al and Andersen et al emphasized that, for a given gene, the stability values reported by geNorm or NormFinder depend on the genes with which it is compared, and that co-regulations could lead to falsely good stability results [9,20]. Thus, results provided by these software programs should be considered as trends and should probably not be generalized without proper validation.

Second, we tested the inter-group stability by looking at possible differential regulation of our 12 candidates between clinically relevant subgroups: HNSCC versus normal matched mucosa, T1-T2 versus T3-T4 and N0 versus N+. Theoretically, the expression level of a reference gene should not be influenced by the experimental conditions, and some authors consider such regulated genes as irrelevant for normalization [15]. In our study, we found that nine out of 12 reference genes were differentially expressed between HNSCC and normal mucosa. This result was not in accordance with the good stability values obtained not only with geNorm but also with NormFinder, which is supposed to take into consideration the inter-group comparison. To resolve this ambiguity, a distinction between statistically and clinically different expressions should be made. We think that a regulated gene can be used as a reference gene for normalization, depending on the resolution of the assay. Statistically, regulated reference genes are acceptable for normalization if the magnitude of this regulation is clinically lower than the clinically relevant regulation that is to be measured. For instance, in this study we observed very weak regulations (maximum 1.7-fold) that were statistically significant. From a clinical point of view, these regulations are not actually significant, since most RT-qPCR studies require the detection of at least ten-fold expression differences for clinical applicability Like Huggett et al. and Toegel et al., we recommend the search for reference gene regulations and specification of their magnitude for any expression comparison between two sample groups [4,13]. Once the variation of the reference gene is known, the resolution of the RT-qPCR assay can be determined. Inversely, the choice of reference gene can be determined by the degree of resolution required for the assay. If the goal of a RT-qPCR study is to detect global gene expression differences between two groups, a 1 Log systematic bias introduced by the use of a regulated reference gene is not recommended, but does not preclude the detection of 2 Log regulation of a gene of interest. If the goal is to precisely measure the magnitude of gene expression difference between two groups, even faintly regulated genes should be avoided for normalization.

## Conclusion

Today, a rational basis for choosing reference genes is needed for quantitative expression studies. Here, we present the first study that analyzes the suitability of 12 reference genes in the field of HNSCC. This study offers a large choice of suitable reference genes, among which research teams can choose depending on the specific setting of their experiments.

In tumor tissue and/or normal mucosa, the four best normalization genes are *ALAS, GAPDH, RPS18 *and *SHAD *and the most stable combination of two genes is *GAPDH-SHAD*. *KALPHA-TBP *is recommended for the study of T1-T2 tumors, *RPL27-SHAD *for T3-T4 tumors, *KALPHA-SHAD *for N0 tumors and *ALAS-TBP *for N+ tumors.

*ACT, B2M, GAPDH, HMBS, HPRT, KALPHA, RPS18, RPS29 *and *TBP *are slightly misregulated (<1.7-fold) between tumor and normal mucosa. Nonetheless, they can be used for normalization depending on the resolution required for the assay.

## Methods

### Patients and sample collection

This study was approved by the local ethics committee. Matched pairs of malignant and non-malignant tissue samples were obtained from 46 patients with primary untreated HNSCC, who gave informed consent. All patients were Caucasian and heavy smokers and drinkers. Patient and tumor characteristics are presented in Table 3.

**Table 3:** Characteristics of gene-specific qPCR assays

**Gene name** (synonym)	Genebank Access n° Gene ID	Gene location	Primer sequence 5'-3'	Amplicon size	PCR efficiency
**ACTB** actin, beta	NM_001101 60	7p15-p12	f: tggctggggtgttgaaggtct r: agcacggcatcgtcaccaact	238	1.98
**ALAS1** aminolevulinate delta synthase 1, transcript variant 1	NM_000688 211	3p21.1	f: aacttgccaaaatctgtttc r: ggtgatgagggagtctgaat	159	1.99
**B2M** beta-2-microglobulin	NM_004048 567	15q21-q22.2	f: cagcgtactccaaagattca r: gaatgctccactttttcaat	240	1.95
**GAPDH** glyceraldehyde-3-phosphate dehydrogenase	NM_002046 2597	12p13	f: tgaacgggaagctcactgg r: tccaccaccctgttgctgta	307	1.90
**HMBS** hydroxymethylbilane synthase, transcript variant 1	NM_000190 3145	11q23.3	f: gaaagacaacagcatcatgag r: accaaggagcttgaacatgc	145	1.98
**HPRT1** hypoxanthine phosphoribosyltransferase 1	NM_000194 3215	Xq26.1	f: ctgacctgctggattaca r: gcgaccttgaccatcttt	256	1.90
**K-ALPHA** tuba1a, tubulin	NM_006082 10376	12q13.12	f: cagatgccaagtgacaagac r: tccaacacaaggtcaatgat	257	1.94
**RPL27** ribosomal protein L27	NM_000988 6155	17q21.1-q21.2	f: tcgccaagagatcaaagataa r: ctgaagacatccttattgacg	121	1.94
**RPS18** ribosomal protein S18	NM_022551 6222	6p21.3	f: agcttgttgtccagaccatt r: tgaggaaagcagacattgac	187	1.84
**RPS29** ribosomal protein S29 transcript variant 2	NM_001030001 6235	14q	f: gcactgctgagagcaagatg r: ataggcagtgccaaggaaga	213	1.95
**SDHA** succinate dehydrogenase complex, subunit A, flavoprotein	NM_004168 6389	5p15	f: agcaagctctatggagacct r: taatcgtactcatcaatccg	200	1.80
**TBP** TATA box binding protein	NM_003194 6908	6q27	f: cacgaaccacggcactgatt r: ttttcttgctgccagtctggac	89	2.01

Tissue samples were collected by biopsy during diagnostic endoscopy between April 2005 and April 2007 and were immediately snap frozen and stored in liquid nitrogen (-180°C). The matched non-malignant tissue was collected on the same anatomical site, as far as possible from the primary lesion for tumors crossing the midline and on the opposite side for well-lateralized tumors.

### RNA isolation, quality control and cDNA synthesis

To obtain homogeneous and histologically well-characterized samples for RNA analyses, tissue samples were cut with a cryo-microtome into 50-200 slices of 9 μm thickness in RNase-free conditions. At least three frozen slices taken from the sample core were mounted on glass slides and briefly stained with eosine-hematoxylin for histopathological examination. An experienced pathologist (H.C.) determined the non-malignant or malignant nature of the tissue and specified its composition. HNSCC samples with less than 30% tumor cells were excluded from the study. In addition, normal tissues were controlled and had to be composed of both stroma and its surrounding normal epithelial layer, with no tumor cells to be included in the study. Total RNA was extracted from the remaining tissue slices using an RNeasy Mini Kit (Qiagen, Courtaboeuf, France), following the manufacturer's instructions. RNA quality control and quantification were carried out on an Agilent 2100 Bioanalyzer using Total RNA Nano II Chips (Agilent Technologies, Massy, France). Tissue samples with total RNA with RNA integrity number (RIN) <6 or a concentration <85 ng/μl were excluded.

Subsequently, 1 μg of total RNA was reverse-transcribed using M-MLV reverse transcriptase and oligo dT14-16 as primer (Applied Biosystems, Courtaboeuf, France), following the manufacturer's protocol. Samples were incubated for 10 minutes at 65°C, cooled on ice for 5 minutes, and incubated with reverse transcriptase for 1 hour at 37°C. Reverse transcriptase was then inactivated by heating at 95°C for 5 minutes. The resulting cDNA were eventually diluted 1:10 before being used as PCR template.

### Quantitative real-time PCR (qPCR)

We quantified the mRNA expression of 12 reference genes by real-time RT-PCR using a SYBR Green approach (LightCycler Fast DNA MasterPlus SYBR Green kit) on a LightCycler 480 (Roche, Meylan, France). Stringent primer sets were designed for the 12 reference genes using Oligo 6 Software (MBI, Cascade, CO, USA). To avoid false detection of genomic DNA, although DNase was included in the extraction procedure, amplification was done on spliced regions of the genes. Gene references and primer characteristics are listed in Table 4. For each qPCR reaction, we used 2 μl of the diluted cDNA, 2 μl of 10 μmol.l^-1 ^forward and reverse primer mix, 5 μl LightCycler Fast DNA MasterPlus SYBR Green I and 11 μl PCR Water in a final volume of 20 μl. The PCR cycle conditions were set as follows: preincubation for 10 minutes at 95°C followed by 40 cycles, with each cycle including 15 seconds at 95°C, 15 seconds at 60°C, and 15 seconds at 72°C. The temperature transition rate was 20°C/second. A melting curve was generated by linear heating from 50°C to 95°C in 20 minutes with 10 fluorescence measures every 1°C. Paired malignant and non-malignant samples were always measured in the same run to avoid inter-run variation. One negative control with no template and one positive inter-run control were included for each gene in each qPCR run. All measurements were done in triplicate. Standard curves were generated on separated runs for each gene using 5 serial dilutions (ranging from 1:1 to 1:5000) of the cDNA sample we used as positive inter-run control. As presented in Table 4, these curves displayed efficient amplification (>1.8) for all genes.

**Table 4:** Clinical features of the populations

	**Median**	**Min**	**Max**
**Age**	56.7	41.2	77.7
		**Quantity**	**Percentage %**
**Sex**	Woman	4	8.7
	Men	42	91.3
			
**Site**	Larynx	8	17.4
	Oral cavity	8	17.4
	Hypopharynx	7	15.2
	Oropharynx	23	50
			
**T Stage**	T1-T2	12	26.1
	T3-T4	34	73.9
			
**N Stage**	N0	18	39.1
	N+	28	60.9
			
**M stage**	0	46	100
	1	0	0

Specificity was confirmed by the presence of a single peak at the expected temperature on melting curve analyses.

Crossing point (Cp) values were automatically calculated by the LightCycler 480 ^® ^software using the second derivative method and were imported into qBase software, version 1.3.5, a free program for the management and automated analysis of qPCR data. We used qBase rescaled values for quantitative analyses [25]. Specific gene amplification efficiencies were calculated by qBase from standard curve results and were used in the quantification algorithm. For each gene the inter-assay coefficient of variation in Cp values was <9%.

### Expression stability assessment

We tested the stability of the 12 reference genes by using geNorm version 3.4 and NormFinder version 0.953. These two dedicated software programs, freely available on the internet, are the two most cited tools in the literature for stability assessment of reference genes. The geNorm algorithm calculates a gene expression stability measure M for a reference gene based on the average pairwise variation for that gene with all other tested reference genes [11]. It ranks the stability of candidate genes and gives the most stable combination of two genes. The NormFinder algorithm relies on a model-based estimation variance approach to estimate the overall expression variation of the candidate reference genes, as well as the variation between sample subgroups of the sample set [20], e.g., normal versus cancer samples.

### Statistical analysis

S-Plus 2000 software was used to perform the statistical analyses. The quantitative variables were described by median, minimum and maximum values and the qualitative variables were described by frequencies and percentages. The mRNA expression levels of the 12 reference genes were compared for HNSCC tissue versus normal matched mucosa using a Wilcoxon test for paired data [21]. The Wilcoxon test for unpaired data was used for the expression comparison of T1-T2 versus T2-T3 stages and N0 versus N+ stage. Comparisons were considered significant if p < 0.05. Using a Visual Basic application, we bootstrapped the results provided by geNorm on 10,000 resampled data sets (by random drawing with replacement) extracted from the original set of 96 tissue samples (HNSCC plus normal mucosa) [23].

## Authors' contributions

All authors read and approved the final manuscript. BL was responsible for the conception, design and experimental work of the study and for drafting the manuscript. AE, CR, DJ, FH and SL made substantial contributions to the study conception and design and greatly enhanced the intellectual content of the manuscript. CC contributed substantially to the study design and carried out the statistical analysis. HC was responsible for pathological examination and quality control. GC, CR, OS and JGL carried out the tissue harvesting. JPB supervised the study and approved the final version of the manuscript.
